# Supplementary material for: Integrating contextual sentiment analysis in collaborative recommender systems
Source: PLoS One. 2021 Mar 22;16(3):e0248695. doi: 10.1371/journal.pone.0248695 (PMC7984640; doi:10.1371/journal.pone.0248695)
Supplement: Attachment — Submitted filename: 1st decision-Response to Reviewers Comments (2).docx [file pone.0248695.s001.docx]

**Responses to reviewer’s comments**

Ref.: [PONE-D-20-33161]

Title: **Integrating contextual sentiment analysis in collaborative recommender systems**

**Plos ONE Journal**

*“We wish to thank the anonymous referees for their helpful and constructive comments on a previous version of this paper.”*

Journal Requirements:

| Please ensure that your manuscript meets PLOS ONE's style requirements, including those for file naming. The PLOS ONE style templates can be found at:  <https://journals.plos.org/plosone/s/file?id=wjVg/PLOSOne_formatting_sample_main_body.pdf>  and  <https://journals.plos.org/plosone/s/file?id=ba62/PLOSOne_formatting_sample_title_authors_affiliations.pdf> | Revised accordingly. |
| --- | --- |
| Thank you for stating in your Funding Statement:  "This research is partially supported by the Malaysia Ministry of Education Grant FRGS/1/2014/ICT02/UKM/01/1 awarded to Shahrul Azman Mohd Noah. The funder did not play any role in the study design, data collection and analysis, decision to publish, or preparation of the manuscript."  Please provide an amended statement that declares *all* the funding or sources of support (whether external or internal to your organization) received during this study, as detailed online in our guide for authors at http://journals.plos.org/plosone/s/submit-now.  Please also include the statement “There was no additional external funding received for this study.” in your updated Funding Statement.  Please include your amended Funding Statement within your cover letter. We will change the online submission form on your behalf. | The funding statement has been revised and updated. |
| Thank you for submitting the above manuscript to PLOS ONE. During our internal evaluation of the manuscript, we found some occurrences of overlapping text with the following previous publication(s), some of which you are an author, which needs to be addressed:  - <https://ieeexplore.ieee.org/document/8464694> - <https://ieeexplore.ieee.org/document/7797377> - <https://medium.com/@m_n_malaeb/recall-and-precision-at-k-for-recommender-systems-618483226c54> - <https://www.toptal.com/deep-learning/4-sentiment-analysis-accuracy-traps> - <https://www.rroij.com/open-access/real-time-sentiment-classification-usingunsupervised-reviews.php?aid=48846&view=mobile> - <https://link.springer.com/book/10.1007%2F978-3-319-29659-3>  We would like to make you aware that copying extracts from previous publications word-for-word, particularly outside the methods section, is unacceptable. In addition, the reproduction of text from published reports has implications for the copyright that may apply to the publications.  Please revise the manuscript to quote or rephrase the duplicated text and cite your sources for text outside the methods section.  Please note that further consideration is dependent on the submission of a manuscript that addresses these concerns about the overlap in text with published work. | Citation added  Citation added  rephrased  rephrased  rephrased  Outside the method section. |

Response to reviewer’s comments dated December, 1 2020.

| Reviewers Comment | Revision Justification |
| --- | --- |
| 1. Is the manuscript technically sound, and do the data support the conclusions?  *The manuscript must describe a technically sound piece of scientific research with data that supports the conclusions. Experiments must have been conducted rigorously, with appropriate controls, replication, and sample sizes. The conclusions must be drawn appropriately based on the data presented.*  Reviewer #1: Yes  Reviewer #2: Partly | *Thank you for the valuable comments.* |
| 2. Has the statistical analysis been performed appropriately and rigorously?    Reviewer #1: Yes  Reviewer #2: Yes | *Thank you for the valuable comments.* |
| 3. Have the authors made all data underlying the findings in their manuscript fully available?  *The* [*PLOS Data policy*](http://www.plosone.org/static/policies.action#sharing) *requires authors to make all data underlying the findings described in their manuscript fully available without restriction, with rare exception (please refer to the Data Availability Statement in the manuscript PDF file). The data should be provided as part of the manuscript or its supporting information, or deposited to a public repository. For example, in addition to summary statistics, the data points behind means, medians and variance measures should be available. If there are restrictions on publicly sharing data—e.g. participant privacy or use of data from a third party—those must be specified*.  Reviewer #1: Yes  Reviewer #2: Yes | *Thank you for the valuable comments.* |
| 4. Is the manuscript presented in an intelligible fashion and written in standard English?  *PLOS ONE does not copyedit accepted manuscripts, so the language in submitted articles must be clear, correct, and unambiguous. Any typographical or grammatical errors should be corrected at revision, so please note any specific errors here.*  Reviewer #1: Yes  Reviewer #2: Yes | *Thank you for the valuable comments.* |
| 5. Review Comments to the Author  *Please use the space provided to explain your answers to the questions above. You may also include additional comments for the author, including concerns about dual publication, research ethics, or publication ethics. (Please upload your review as an attachment if it exceeds 20,000 characters)*  Reviewer #1:   a) Discuss Problem Statement clearly in manuscript, discuss more state-of-the-art work, b) Explain performance metrics very clearly and justify, why you used the mentioned performance metrics in the manuscript, need to add more resulting graphs. c) Add a new section named as Future Work, and discuss future work.   Reviewer #2:  This paper describes a recommendation system approach that leverages sentiment analysis to improve performance. The following issues should be addressed before this paper can be considered for publication:   a) Table 1 could be replaced by a Figures with more values of distinct lambda. b) It is not clear if your results are significant better than other competing approaches. c) A better context on recommendation systems should be provided. For example, it is not used only for products. It can be used in wider contexts   See and mention e.g. applications in collaboration and citation analysis  doi: 10.1209/0295-5075/99/48002 doi:10.1007/s00799-020-00288-2   d) Figure quality should be much improved. e) There is no need to define cosine similarity. f) It would interesting to mention that other strategies for recommendation system could also benefit from the proposed strategy. For example, graph based approaches could benefit from the proposed sentiment analysis in recommendation systems. See and mention e.g.   doi: 10.1016/J.INS.2018.02.047 doi: 10.1145/544220.544231  for strategies that could be combined and benefit from your approach.   g) TAbles (in figure) should be provided in a standard (e.g. latex) way, and not in a microsoft office figure. h) Perspectives for future works should be provided. Which other works or lines of research could benefit from this strategy? | *Thank you for the valuable comments.*  Responses to Reviewer #1:   a. Problem statement and state-of-the-art work were further elaborated in the Introduction section. b. Explanation of the used performance metrics were further elaborated and subsequently justified in the Experiments and Results section. c. A new section named Future Work is included in the revised version of the paper   Responses to Reviewer #2:   a) Noted. Table 1 has been replaces by a figures with more values of distinct lambda ranging from 0.2 till 0.8 to indicate more clear and specific results. b) Results of significant test were included in Table 5 and 6. The results are significant better that the other two baselines which is traditional recommender system and traditional sentiment based recommender system. It has been proven in the experiments by comparing the results using the same format, same domain and same number of test sets. c) In this experiment, we demonstrated in a wider context not only focus in one domain. We also use for movie and films domain not only for products. Hence, it can be used in different applications as well by performing in other domain such as medicine and political domains (See Future Works section). d) The quality of figures have been improved. e) The definition of Cosine similarity definition has been removed. f) Other strategies that can be benefit from the proposed approach as recommended by the 2^nd^ reviewer is included in the Discussion & Conclusion section. g) Tables (in figures) has been changed in latex format. h) Prospective for future work has been added in the Future Work section. |
